# Assessment of spirometric decline from silica dust: threshold values and calculation methods for cumulative exposure

**DOI:** 10.1186/s12995-026-00507-7

**Published:** 2026-03-25

**Authors:** Christin Schröder, Dennis Nowak

**Affiliations:** 1 Federal Institute for Occupational Safety and Health (BAuA), Division “Work and Health”, 10317 Berlin, Germany; 2 Institute and Clinic for Occupational, Social and Environmental Medicine, LMU University Hospital, Comprehensive Pneumology Center (CPC) Munich, German Center for Lung Research (DZL), Munich, Germany

## Abstract

**Background:**

This study investigates the association between crystalline silica dust exposure and lung function decline. Understanding whether exposure-response relationships include threshold values is critical for contributing to workplace health protection. We compared four mechanistically different models to determine which best characterizes this association.

**Methods:**

Data from 1,418 workers from the Wismut German Uranium Mining Cohort Study from 1970 to 1991 with 7,116 spirometry measurements were analysed. Cumulative exposure was calculated using a job exposure matrix. Four models based on different pathophysiological mechanisms were compared: (1) constant annual threshold without overload, (2) constant annual threshold with overload, (3) delayed onset (cumulative threshold only), and (4) dynamic threshold decreasing over time.

**Results:**

Models 1, 3 and 4 showed a critical annual exposure value of approximately 0.09-0.10 mg/m^3^ (the concentration below which no harmful accumulation occurs). Model 2 had convergence problems due to mathematical discontinuity. Model 1 demonstrated the biologically most plausible relationship between threshold exceedance and spirometric decline, with a cumulative threshold of 2 mg/m^3^. This model predicts clinically significant functional decline within realistic occupational timeframes (e.g., 10 years at 0.3 mg/m^3^ exposure).

**Conclusion:**

The results confirm that high silica dust exposure leads to an accelerated decline in lung function. Model 1 appears to be the most appropriate, despite limitations such as limited age range and sole focus on spirometry. The results demonstrate an association between cumulative silica dust exposure above approximately 0.09 mg/m^3^ and accelerated spirometric decline. Model 1 (constant annual threshold) provides the most consistent and biologically plausible results. These threshold estimates have high relevance in occupational health settings aimed at preventing workers exposed to silica dust from lung function decline. Study limitations include the limited follow up and lack of data on individuals reaching the cumulative threshold. The majority of included persons were smokers (84%), which also affected lung function.

## Background

Exposure to crystalline dusts is implicated in the development of various diseases. These particles are primarily found in soil and sand, with quartz—the crystalline form of silica—being the most abundant. Because quartz is so widespread, many people encounter it during their working lives, for example when working in quarries or the mining and manufacturing industry.

It is well established that exposure to silica dust can cause or promote several diseases, such as lung cancer, silicosis, silicotuberculosis and chronic obstructive pulmonary disease (COPD). COPD is characterized by persistent narrowing of the airways and progressive impairment of lung function. Globally, COPD presents a high disease burden and causes 3.5 million deaths per year, according to the World Health Organization [1]. Although a significant proportion of COPD cases can be attributed to smoking and indoor biomass burning, exposure in the workplace is another important risk factor [2].

For example, according to the Occupational Safety and Health Administration (OSHA), 2.3 million workers in the United States are exposed to silica at work. In the European Union, an estimated 5.3 million workers are similarly exposed, and in Germany specifically, 628,021 workers were exposed in 2011 [3].

Various studies have shown that occupational exposure to silica dust contributes to COPD [4–6]. In addition, there is a study which described exposure related changes in lung function [7]. Importantly, these associations have been observed independently of radiographic evidence of silicosis or other parenchymal changes, suggesting that silica dust affects airway function through mechanisms distinct from fibrotic lung disease [4].

Understanding these mechanisms requires consideration of how the lung normally clears inhaled particles. Pulmonary clearance occurs via three distinct mechanisms:

- mucociliary clearance in the conducting airways, where particles are captured in the mucus layer and transported out of the lungs within hours [8]
- alveolar macrophages in the peripheral lung, which phagocytose deposited particles [9]
- translocation, which allows small particles (<100 nm) to cross the alveolar-capillary barrier and enter the interstitium, lymphatics, or bloodstream.

Crystalline silica poses a unique challenge to these clearance mechanisms due to its cytotoxic effects on alveolar macrophages [9]. Unlike inert particles, silica causes macrophage cell death following phagocytosis. Since alveolar macrophages maintain their population through local self-renewal rather than recruitment from the bone marrow, silica-induced macrophage death can exceed the capacity for local replacement. This creates a biological basis for a threshold effect: below a certain exposure level, macrophage proliferation can compensate for particle-induced cell death, maintaining effective clearance; above this threshold, net macrophage loss leads to impaired clearance, particle accumulation, and chronic inflammation. When dust concentrations become excessively high, the lung experience a so-called “overload” effect, where the natural defence mechanisms are overwhelmed completely [9]. An initial analysis by Möhner et al. [10] investigated this issue, providing preliminary evidence of a link between quartz dust exposure and COPD, suggesting the existence of an exposure threshold. This phenomenon of “overload” has also been described in detail by Morrow [11].

It is essential to understand whether a threshold exists for silica-induced lung damage in order to protect the millions of workers exposed to it globally. Identifying such a threshold could contribute to the development of evidence-based occupational exposure limits and prevention strategies.

The present study builds on the findings of [10] and extends them by applying two additional models. Thus, a total of four models are compared to quantify the association between silica dust exposure and spirometric and to elucidate any dose – response relationships that may emerge. The aim is to answer the question which of the models best describes excessive decline in spirometry caused by silica dust and in particular whether there is a threshold value.

## Method

This analysis is based on data from the Wismut German Uranium Mining Cohort Study. The cohort examined here is a sub-cohort of the larger German uranium miner cohort. Only employees who started working underground after 1970 were considered. The reason for this was that the working conditions before and after 1970 differed considerably and exposure to quartz dust also steadily decreased. The last measured values evaluated were from 1991.

### Exposure data

Cumulative exposure to respirable crystalline silica (RCS) was calculated using a comprehensive job-exposure matrix (JEM) developed specifically for the Wismut cohort [10]. The JEM assigns exposure estimates based on job title, calendar period, and work location. Exposure data were derived from approximately 1.3 million personal and stationary dust measurements collected by the German Social Accident Insurance (DGUV) during the operational period of the mines.

The JEM accounts for temporal trends in exposure levels resulting from improved ventilation and dust suppression measures implemented over time. For each worker, annual mean RCS concentrations (in mg/m^3^) were estimated based on the job categories held during that year. Cumulative exposure was then calculated as the sum of annual exposures, expressed in mg/m^3^ -years.

The validity of the JEM has been evaluated in previous publications, demonstrating good agreement between estimated and measured exposure levels for workers with personal monitoring data [10].

### Lung function data

Lung function data were gathered from the workers at the start of their employment and subsequently every two years. These examinations took place in the mornings, prior to the work shift, and were carried out using a Stollberg spirometer. Pulmonary function testing (PFT) was performed without the administration of a bronchodilator. From the measured forced expiratory volume in one second (FEV₁) and forced vital capacity (FVC), we calculated the z-score of the FEV₁/FVC ratio using the Global Lung Function Initiative (GLI) 2012 reference equations [12]. The GLI-2012 equations account for age, sex, height, and race, enabling precise determination of the lower limit of normal (LLN), defined as z = −1.645. The z-score represents the number of standard deviations by which an individual’s observed value differs from the predicted value for a healthy reference population of the same age, sex, height, and race. A z-score of zero indicates that the observed value equals the predicted mean; negative z-scores indicate values below the predicted mean.

Data were described in detail in Kreuzer et al. [13].

### Statistical models

We evaluated four statistical models based on different pathophysiological assumptions regarding the relationship between silica exposure and lung function decline. All models aim to identify a threshold exposure level above which accelerated lung function decline occurs. The key difference between the models lies in how this threshold is conceptualized and how cumulative exposure above the threshold is calculated.

**Fig. 1:**
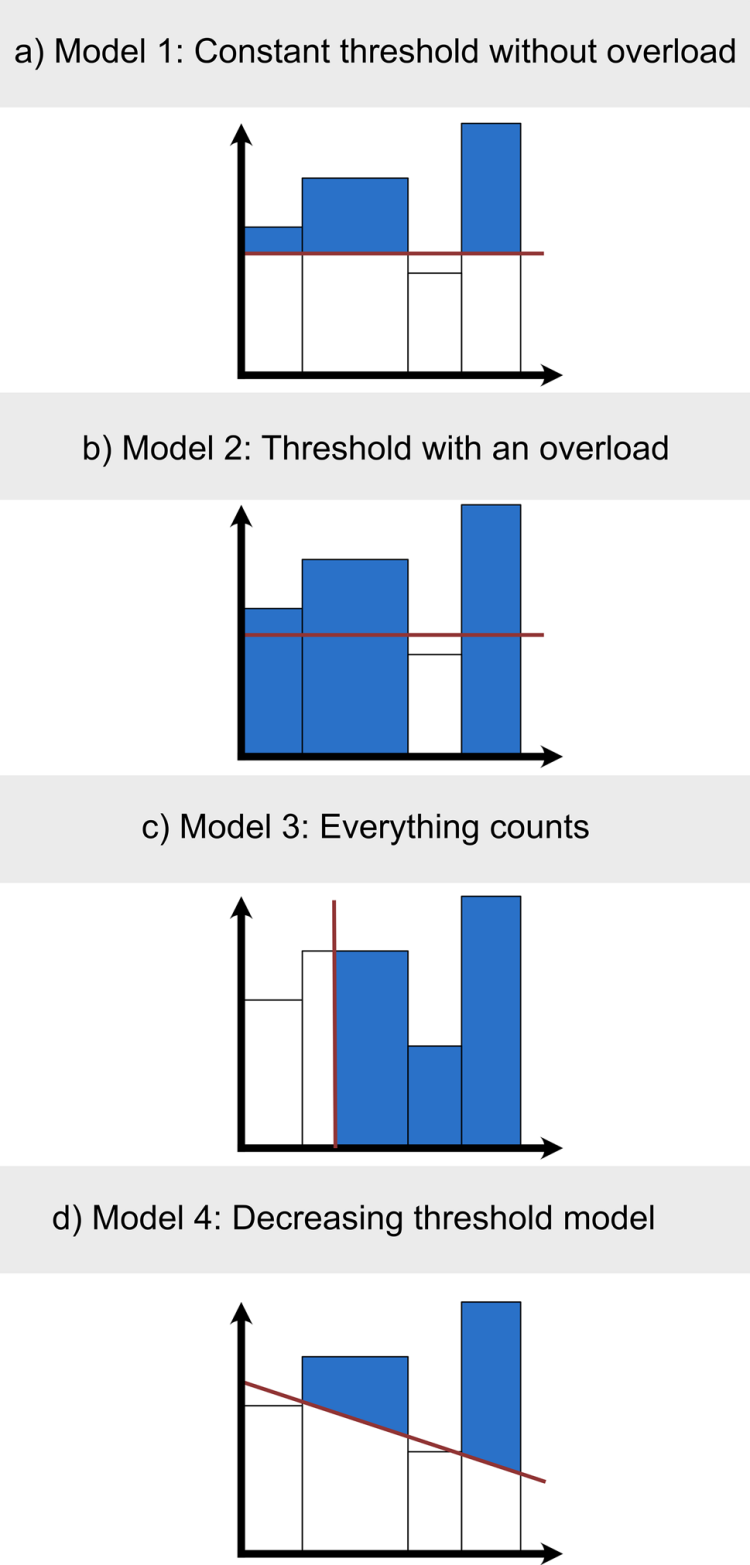
Schematic illustration of the four threshold models (**a-d**). (**a**) constant threshold without overload, (**b**) constant threshold with overload, (**c**) delayed onset, (**d**) dynamic threshold. Shaded areas indicate exposure contributing to cumulative harmful dose. Mathematical formulations are provided in the methods section

All models are described in detail in the text below, and their concepts are visually represented in Fig. 1. Each rectangle indicates exposure over a specific time frame, while the line represents the modeled threshold. The blue area denotes accumulated exposure levels and corresponds to the hypothesized dose that impacts lung function.

Model 1 and a linear model were already compared and published [10].

Model 1 is based on the assumption that the lungs have a limited clearance capacity, primarily mediated by alveolar macrophages [14]. Oberdörster [15] demonstrated that clearance mediated by alveolar macrophages is impaired when exposure to particles exceeds approximately 1 µl per gram of lung tissue. Below this threshold, particles can be effectively cleared without causing cumulative damage. This is what Model 1 represents.

In other words, this model is based on the hypothesis that the lungs can tolerate exposure up to a certain level without experiencing adverse effects. However, once this threshold is exceeded, each additional unit of exposure contributes to progressive deterioration in lung function.

The model assumes a fixed exposure threshold (τ) above which exposure to silica contributes to a decline in lung function. As Fig. 1a shows, only the portion of annual exposure that exceeds τ contributes to the cumulative harmful dose each year. The cumulative exposure metric is calculated as follows:

$$C{E_1} = \mathop \sum \limits_{t = 1}^T {\rm{max}}({E_t} - \tau ,0)$$

where is the $$ {E_t}$$ annual exposure in year $$t$$, $$\tau $$ the threshold parameter to be estimated and T the total number of years worked, respectively.

Model 2 (Fig. 1b) builds on the principles of Model 1, additionally incorporating complete clearance failure when the threshold value is exceeded. Past research has shown that under conditions of particle overload, the clearance system can become overwhelmed, leading to a qualitative change in particle processing [9, 16]. In this scenario, even particles that would normally be broken down contribute to lung overload because the clearance system fails completely. This is consistent with the observation that macrophages heavily loaded with dust lose their mobility and phagocytic function [17].

Model 2 is therefore based on the hypothesis that years of high exposure lead to complete clearance failure, whereas years of low exposure allow normal clearance.

This model also assumes a fixed annual threshold but incorporates an ‘overload mechanism’. If annual exposure in a given year exceeds this threshold, the entire annual exposure (not just the excess) contributes to the cumulative harmful dose. Years with exposure below the threshold do not contribute. The cumulative exposure is:

$$C{E_2} = \mathop \sum \limits_{t = 1}^T {E_t} \cdot 1({E_t} > \tau )$$

where $1({E_t} > \tau )$ is an indicator function equal to 1 if ${E_t} > \tau$and 0 otherwise, ${E_t}$ is the annual exposure in year $t$ and $\tau$ is the threshold parameter to be estimated.

Model 3 (Fig. 1c) is based on the observation that silica-related lung diseases usually only occur after a latency period following initial exposure. Epidemiological studies have shown that silicosis and associated lung function impairments often occur years or even decades after initial exposure. This suggests that a certain cumulative dose of exposure to silicon dioxide must be reached before clinically detectable damage occurs [18]. This latency period may reflect one or more of the following: (1) sufficient particle accumulation to overcome local defence mechanisms, (2) the development of chronic inflammation, or (3) the initiation of fibrotic processes. Model 3 examines whether there is a threshold level of cumulative exposure that must be exceeded before a measurable decline in lung function begins.

This model assumes that a decline in lung function only begins after a certain cumulative exposure level has been reached. Unlike Models 1, 2 and 4, Model 3 estimates a threshold for total cumulative exposure rather than annual exposure. The effective cumulative exposure is:

$$C{E_3} = {\rm{max}}\left( {\mathop \sum \limits_{t = 1}^T {E_t} - {\tau _{cum}},0} \right)$$

where ${\tau _{cum}}$ is the cumulative threshold parameter and ${E_t}$ is the annual exposure in year $t$.

Model 4 (Fig. 1d) incorporates the hypothesis that prolonged exposure progressively impairs the lung’s clearance capacity. Animal studies have demonstrated that exposure to silica particles can result in structural damage to the mucociliary apparatus, including cilia that are shorter and disorganised, as well as ultrastructural abnormalities in ciliated epithelial cells [19]. Furthermore, silica is directly cytotoxic to alveolar macrophages [20], which could lead to a progressive reduction in the number and function of these cells with ongoing exposure. Model 4 tests whether the threshold decreases linearly over time, reflecting cumulative damage to clearance mechanisms.

It builds upon Model 1 by permitting the threshold to decrease over time, thereby reflecting the notion that prolonged exposure can gradually impair the lung’s clearance capacity. The threshold in year t is modelled as follows:

$${\tau _t} = {\tau _0} - \beta t$$

where τ₀ is the initial threshold and β is the rate of decrease per year. The cumulative exposure is then:

$$C{E_4} = \mathop \sum \limits_{t = 1}^T {\rm{max}}({E_t} - {\tau _t},0)$$

where $${E_t} $$ is the annual exposure in year $$t, {\tau _t} $$ is the threshold parameter in year t.

### Statistics

The analysis followed a two-stage procedure to determine exposure thresholds and quantify their health effects. All analyses were conducted in R (version 4.2) [12].

#### Stage 1: estimation of threshold values

In the first stage, we used Bayesian regression methods to estimate the threshold values. For Models 1, 2, and 4, we estimated the annual exposure threshold (τ or τ₀)—the level below which no damage accumulates. For Model 3, we estimated the cumulative threshold (${\tau _{cum}}$)—the total exposure needed before damage becomes measurable.

The Bayesian models were implemented using the brms package [13] and STAN [14]. To ensure the estimated thresholds stayed within realistic ranges, we constrained them using an inverse-logit transformation scaled to the observed exposure range.

The statistical calculations used four independent Markov chains, each with 8000 iterations. The first 2000 iterations in each chain served as a “warm-up” period and were discarded, leaving 6000 iterations per chain for the final estimates. Convergence was assessed using the potential scale reduction factor (R) and effective sample size (ESS).

#### Stage 2: estimation of cumulative exposure effects

Once the threshold values were estimated in Stage 1, we used them to calculate each individual’s cumulative exposure according to the respective model formulas. These cumulative exposure values were then analyzed in the second stage using linear mixed-effects models (LME) to quantify the relationship between cumulative exposure and lung function decline. Thereby we estimated the cumulative dose required to obtain the lower limit of normal (LLN), which is defined as z-score = −1.645. This z-score is widely recognised as the threshold for clinically significant lung function impairment.

The LME models included a random intercept for each individual to account for repeated measurements over time. Smoking status was included as a covariate. This second stage allowed us to determine the cumulative exposure level at which a clinically significant decline in lung function occurs.

### Model comparison and validation

To compare the four models and determine which best fits the data, we used leave-one-out cross-validation (LOO). This method evaluates how well each model predicts data that was not used to fit the model. The model with the lowest LOO value was considered to have the best predictive performance.

For Model 4, we additionally estimated the rate of threshold decline over time (slope k). This parameter was constrained to be zero or negative, allowing the threshold to decrease or remain constant, but not increase.

All results from Stage 1 are reported with 95% credible intervals.

## Results

**Fig. 2:**
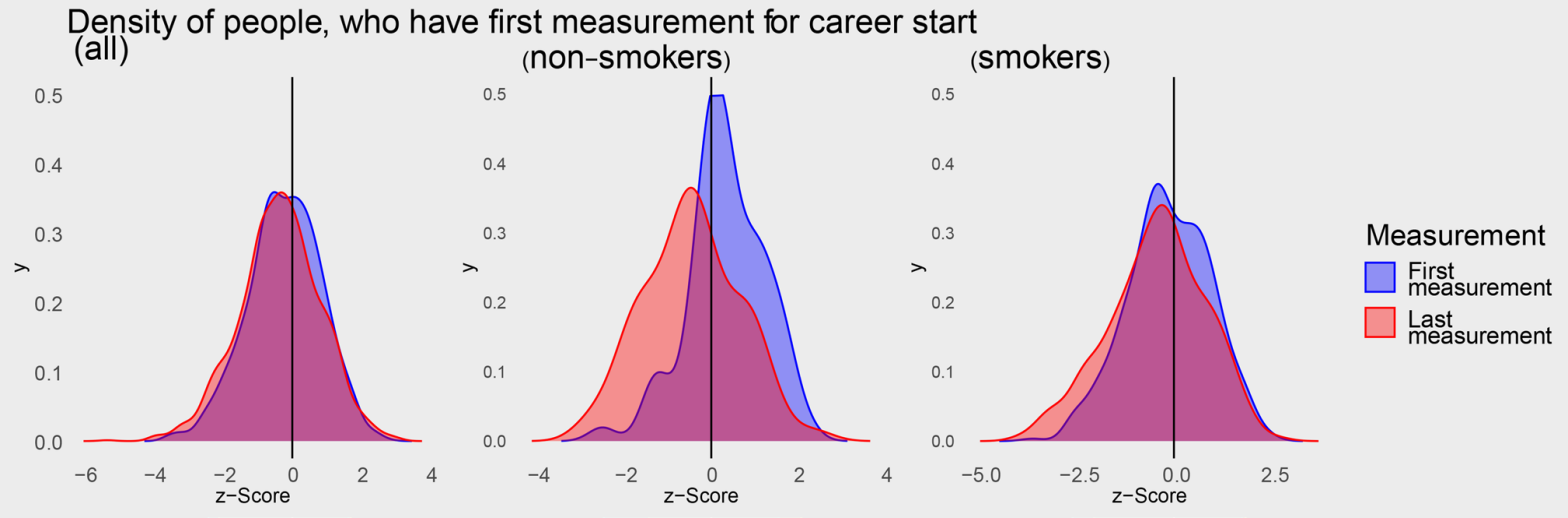
Density function of the first spirometry and the last measurements for the entire workforce (left) and then separately for non-smokers/smokers (middle/right)

The dataset contains data from 1418 men. Median number of spirometric measurements per person in the dataset is five. In total, 7116 spirometry measures were analysed (Table 1). From 1976 to 1988, over 400 spirometry measures were taken each year and peaked in 1985 with 559 measures. The lowest z-score was −5.32 and the highest 3.08. The median z-score decreased from the first measurement to the last measurement from −0.19 to −0.34 over all persons, which corresponds to a reduction from the 42nd percentile to the 36th percentile. The shift can also be seen in Fig. 2. The decrease in the z-score is particularly apparent in non-smokers. At least five measurements were carried out on 721 people. The median age at the first measurement was 22 years with an interquartile range (IQR) [20,26] and the median age at the last measurement was 33 years with IQR [29,34].

**Table 1:** Description of data set

Persons (N)	1,418
number of spirometry measurements (total)	7,116
Spirometry measurements per person (Md; [IQR])	5 [3, 7]
Age (Md [IQR])	27 [23, 31]
Smoker (N [%])	1,187 (84%)
Years of employment in this company	12 [7, 15]
Concentration of silica dust per year in mg/m^3^ (Md, [IQR])	0.06 [0.03, 0.11]
Concentration of silica dust at first exposure measurement in mg/m^3^ (Md, [IQR])	0.01 [0.00, 0.05]
Concentration of silica dust at last exposure measurement in mg/m^3^ (Md, [IQR])	0.06 [0.03, 0.10]
Concentration of silica dust cumulative exposure measurement in mg/m^3^ ⋅ year (Md, [IQR])	0.64 [0.28, 1.13]
z-Score of lung function (Md, [IQR])	−0.36 [−1.06, 0.36]
z-Score of lung function at first measurement (Md, [IQR])	−0.19 [−0.89, 0.52]
z-Score of lung function at last measurement (Md, [IQR])	−0.34 [−1.05, 0.40]

Models 1, 3 and 4 came to a clear result, model 2 did not (see Table 2). Model 2 showed convergence problems, which is reflected in several different results for the threshold value. The cause of this lies in the discontinuity of the function. Even inserting a very strongly decreasing function at the point of discontinuity did not solve the convergence problems. Models 1 and 4 showed similar results for the threshold. The slope of the threshold line for Model 4 has such a small value (0.000286) that it asymptotically appears to be a straight line with no slope. The model 3 threshold is so low that almost every annual workplace exposure is above the threshold. Only a tiny fraction of the cumulative exposure is subtracted.

**Table 2:** Estimated threshold values and regression coefficients for the four models with 95% credible intervals [in brackets]

	Model 1	Model 2	Model 3	Model 4
Threshold τ (mg/m^3^/year)^a^	0.09	0.10 0.115 0.14	0.015	0.10–0.000286*t
Smoking (yes vs. no)^b^	−0.25 [−0.37, −0.13]	−0.24 [−0.36, −0.12]	−0.24 [−0.36, −0.12]	−0.26 [−0.38, −0.14]
Cumulative exposure above threshold^c^ (mg/m^3^ ⋅ year)	−0.77 [−0.97, −0.56]	−0.23 [−0.30, −0.17]	−0.15 [−0.21, −0.10]	−0.74 [−0.96, −0.52]
Leave-One-Out IC (SE)^d^	3982.50 (0.74)	3984.21 (0.75)	3990.15 (0.75)	3983.39 (0.75)

^a^ Annual exposure threshold above which lung damage is expected to occur

^b^ Regression coefficient for smoking status; values in brackets represent 95% credible intervals

^c^ Regression coefficient per unit cumulative exposure above threshold; values in brackets represent 95% credible intervals

^d^ Leave-One-Out Information Criterion for model comparison; lower values indicate better fit

The models were compared using the leave-one-out method which showed that the lowest value was determined for model 1, making it the best model.

Depending on the model, different times are required to have a significantly increased risk of impaired lung function. The fewest years to achieve a significant reduction in lung function were needed with model 1 when there was a high mean annual load (Table 3).

**Table 3:** Years of exposure required to reach the cumulative threshold at different mean annual silica dust concentrations

Cumulative threshold		Modell 1	Modell 2	Modell 3	Modell 4
		2 mg/m^3 ⋅ year^	7 mg/m^3 ⋅ year^	11 mg/m^3 ⋅ year^	2 mg/m^3 ⋅ year^
concentration	0.025	-	-	440	-
	0.061	-	-	180.5	-
	0.105	400	66.7	104.9	Similar to model 1
	0.250	13.3	28	44.06	Similar to model 1
	0.300	10	23.3	36.7	Similar to model 1

## Discussion

It was possible to implement all four conceivable models for lung function decline with occupational silica dust exposure. However, Model 2 had calculation issues, and its uniqueness could be improved by introducing a steeply declining function at the discontinuity point.

Previous literature has noted that exposure to various inhaled agents reduces mucociliary clearance [8]. Animal studies have shown a similar phenomenon for silica dust [19]. In Model 4 of our approaches, decreasing clearance over time was modelled via a declining linear function; however, the slope was so minimal that it was essentially equivalent to a straight line with no slope. The discrepancy between our findings and animal models may have multiple causes—species-specific physiological differences, varying exposure levels, controlled environments, and potential confounders including smoking. Another critical factor could be the healthy worker effect.

In Model 3, the time to onset of damage was so short that deterioration in lung function would effectively begin with the start of employment. However, according to that model, a worker would have to remain in the occupation for an unrealistically large number of years before any statistically significant impact would manifest and the model had the highest value in model comparison with leave one out.

By contrast, Model 1 appears to be the most suitable for estimating lung function under occupational exposure. Under Model 1, individuals experiencing high average annual concentrations of silica dust face an increased risk of decline in lung function toward the end of their working years. Moreover, even a realistic duration of employment would suffice to produce a measurable decline in lung function for those with very high exposures.

No interaction between smoking and silica dust exposure was modelled. Although the literature suggests that there may be combined effects, occupational exposure limits are the same for all workers. Our threshold value of 0.09 mg/m^3^ is based on a cohort in which 84% of individuals were smokers and it represents a value for typical occupational groups, which might be a conservative estimation.

No participant reached the cumulative exposure thresholds estimated by our models. Our analysis provides two threshold estimates: the annual exposure threshold (τ ≈ 0.09 mg/m^3^) and the cumulative threshold (2 mg/m^3^ ⋅ year for Model 1). The annual threshold is well-supported by observed data, as workers had exposures both above and below this value. However, the cumulative threshold involves extrapolation beyond our observed range. While we observe a clear dose-response relationship within our data, we cannot directly validate the predicted cumulative threshold. Therefore, the annual threshold provides a robust basis for exposure control, while cumulative thresholds should be considered preliminary estimates requiring validation in cohorts with longer follow-up.

As mentioned above, these are the first analyses of a threshold value. Therefore, no other analyses are available for comparison. However, using a different method, Möhner and Nowak (2020) come to the same conclusion as this analysis. The results for the cumulative exposures and the point at which a deterioration in health can be expected are consistent with other studies. In Wardyn [21], a group of workers with less than 1 mg/m^3^ per year silica dust exposure is described and compared to workers who were exposed to at least 1 mg/m^3^ ⋅ year. It is striking that the group with the low exposure did not show a deviation of the z-score (−0.042, CI (−0.178, 0.095)), while the highly exposed group showed a deterioration of −0.338 (−0.702; 0.025). When looking at the z-values for the GLI-2012 score, the decline in the exposed group is even more pronounced. Unfortunately, only a rough classification into these two groups was made so that a detailed comparison was not possible.

Despite these findings, our study has several limitations. Firstly, the data set is limited to measurements taken between 1970 (at the earliest) and 1991 (at the latest), meaning that only data with a limited occupational history is available. The highest age at which measurements were obtained was 35 years. Second, we modelled only lung function data based on spirometry; other variables (e.g., comorbidities) were not considered. Furthermore, none of the participants in this cohort actually reached the cumulative dose threshold required to validate the models fully. Thus, we could not confirm how accurately these models perform beyond the observed exposure range. When extrapolating working conditions, only in Model 1 did certain individuals eventually reach the cumulative exposure needed to induce a significant decrease in lung function.

The advantage of the study is that multiple measurements are available for individual persons over a period of years and exposures are not only available as categories.

## Conclusion

This study shows that cumulative exposure to silica dust is associated with accelerated decline in lung function. Among the four evaluated models, the constant threshold model overload (Model 1) provided the most consistent results, identifying a threshold value of 0.09 mg/m^3^. These findings support the existence of an exposure threshold for silica dust to decline the lung function.

## Data Availability

At time of data collection, participants agreed in analysis and presentation of aggregated data but not in publication of individual data. So, the data are not publicly available due to legal restrictions.
